# Strand with mutagenic lesion is preferentially used as a template in the region of a bi-stranded clustered DNA damage site in *Escherichia coli*

**DOI:** 10.1038/s41598-020-66651-0

**Published:** 2020-06-16

**Authors:** Naoya Shikazono, Ken Akamatsu

**Affiliations:** Institute for Quantum Life Science, National Institutes for Quantum and Radiological Science and Technology, 8-1-7 Umemidai, Kizugawa, Kyoto, 619-0215 Japan

**Keywords:** Genomic instability, Base excision repair, DNA synthesis

## Abstract

The damaging potential of ionizing radiation arises largely from the generation of clustered DNA damage sites within cells. Previous studies using synthetic DNA lesions have demonstrated that models of clustered DNA damage exhibit enhanced mutagenic potential of the comprising lesions. However, little is known regarding the processes that lead to mutations in these sites, apart from the fact that base excision repair of lesions within the cluster is compromised. Unique features of the mutation frequencies within bi-stranded clusters have led researchers to speculate that the strand containing the mutagenic lesion is preferentially used as the template for DNA synthesis. To gain further insights into the processing of clustered DNA damage sites, we used a plasmid-based assay in *E. coli* cells. Our findings revealed that the strand containing a mutagenic lesion within a bi-stranded clustered DNA damage site is frequently used as the template. This suggests the presence of an, as yet unknown, strand synthesis process that is unrelated to base excision repair, and that this process plays an important role in mutagenesis. The length of the region of strand preference was found to be determined by DNA polymerase I.

## Introduction

DNA damage induced by ionising radiation has the potential to initiate events that can lead ultimately to various biological consequences including mutations, chromosomal aberrations and cell death. Various types of DNA damage, such as base lesions, apurinic/apyrimidinic (AP) sites, strand breaks, interstrand crosslinks and DNA-protein crosslinks, are produced by ionising radiation. Among these diverse types of DNA damage, clustered DNA damage, which is defined as two or more DNA lesions within one to two helical turns of DNA, has been proposed to be strongly relevant to the biological consequences of ionising radiation^1,2^. The biological significance of clustered DNA damage has been extensively studied, mainly with the use of chemically synthesized DNA lesions^3–6^. Various types of synthetic base lesions, such as 7,8-dihydro-8-oxoguanine (8-oxoG), thymine glycol (Tg), dihydrothymine (DHT), and 5-hydroxyuracil (hU), as well as AP sites and single-strand breaks (SSB), have been subjected to analysis.

In general, base lesions and AP sites are repaired via the base excision repair (BER) pathway^7^. In *Escherichia coli* (*E. coli*), a glycosylase, such as Fpg or Nth, initially recognizes the base lesion and excises the damaged base leaving an AP site. The AP site is further processed by an AP endonuclease (ExoIII or NfoI) or the AP-lyase activity of the glycosylase, thereby generating an SSB. A single deoxyribonucleotide (nt), in the case of short-patch repair, or multiple nt, in the case of long-patch repair, are then incorporated by DNA polymerase I (PolI) from the end of the SSB, and finally, the breaks are sealed by DNA ligase. *In vitro* studies have revealed that, although single, isolated base lesions/AP sites are repaired efficiently, the repair of lesions within a cluster is often retarded depending on the type, position and number of lesions within a cluster^3,4^. Consistent with these *in vitro* results, several groups have found an increase in mutation frequency (MF) within clustered DNA damage sites with two or more lesions in various configurations^8–16^. Studies of MF within these clustered DNA damage sites have indicated that: 1) there is a preferential order in the excision of different types of base lesions, 2) the resultant SSB generated after the initial excision of a lesion retards the repair of a nearby mutagenic lesion on the complementary strand at the time of replication, and 3) eventually the SSB is either repaired or tolerated to allow replication of the damaged DNA.

Apart from this retardation of repair of the mutagenic lesions, the molecular mechanism of mutation induction within clustered DNA damage sites is still largely unknown. Some of the unique features of the MF in bi-stranded clusters, are: 1) the MF of 8-oxoG within a bi-stranded cluster is remarkably higher than that of a single 8-oxoG in the *fpg mutY* strain of *E. coli*, a strain in which the excision of 8-oxoG is considered to be minimal^10–12,15,17^; 2) the MF within a 8-oxoG+DHT bi-stranded cluster from a transformed clone is, in some cases, over 50%^11^; 3) the MF of hU within a three-lesion cluster is over 90%^14^; and 4) the presence of a persistent SSB at a distance of ~20 nt in either the 5′ or 3′ orientation increases the MF of a single 8-oxoG^18^. These observations led us and others to suggest that the mutagenic process most likely involves the preferential use of the DNA template containing the mutagenic lesion. It was speculated that a persistent SSB within a clustered DNA damage site leads to replication impairment and that the strand opposite the mutagenic lesion is likely lost.

When the template DNA is interrupted by an SSB, the replication fork may collapse and result in strand breakage^19,20^. Indeed, it has been suggested that a persistent SSB within a non-DSB cluster results in the production of a replication-dependent double-stranded (ds) DNA end^21^. In addition, rather than releasing dsDNA ends by fork collapse, the dsDNA end can be processed during the remodelling of the stalled replication fork, which potentially involves multi-step processes such as lesion skipping, fork reversal and/or template skipping^22–24^. Homologous recombination proteins appear to play a critical role in recovery from replication impairment^20,25^. In prokaryotes, the key protein in the recovery of replication stalling or collapse is RecA^20^. Another important process to consider when exploring the underlying mechanism of template strand preference is DNA synthesis. PolI, which is known to operate in BER, strongly suppresses the mutagenic potential of 8-oxoG in a bi-stranded cluster, and thus, is proposed to play an important role in the mutagenesis of clustered DNA damage sites^13^. However, it is still unclear how PolI reduces mutation induction, as previous studies strongly suggest that BER is compromised within clustered DNA damage sites.

To study the template strand preference in the region of a clustered DNA damage site, we previously developed a plasmid-based assay in *E. coli*^26^. It was found that the proportions of the two DNA strands used as the template were unaffected by the presence of a clustered DNA damage site ~1,400 nt away. This result demonstrated that the strand opposite the mutagenic lesion of the plasmid was not totally lost during replication. However, template strand preference around a clustered DNA damage site has not been directly shown, and little is known regarding the range of strand preference or the underlying mechanisms involved. Here, we examined whether: 1) the strand is displaced and synthesized around a bi-stranded cluster, and 2) the strand preference is affected by RecA and/or PolI. We found that a characteristic strand bias is observed around uracil + 8-oxoG cluster and that PolI, but not RecA, primarily determines the length of the region of template strand preference.

## Results

**Figure 1:**
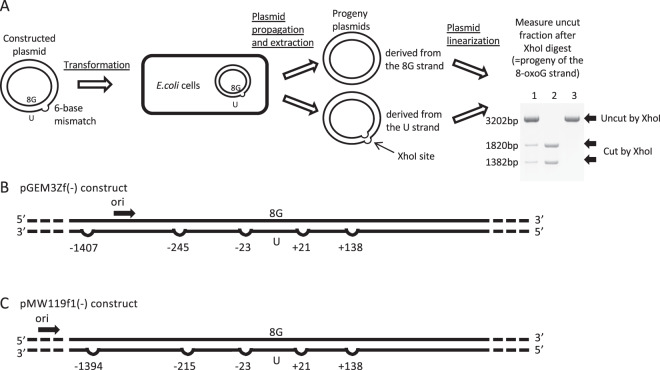
Schematic representations of the experiment and plasmids used to measure the level of strand bias surrounding a clustered DNA damage site. (**A**) Outline of the experiment. A plasmid was constructed that harboured a bi-stranded clustered damage site comprising uracil (U) and 8-oxoG (8G), as well as a 6-nt mismatch (XhoI site) used as a strand-specific marker. The constructed plasmids were introduced into *E. coli*, and the propagated plasmids were subsequently extracted. The level of strand preference was determined by digestion of the plasmids with XhoI, which cut the plasmids that were the progeny of the strand with the XhoI site (uracil-containing strand). XhoI-digested plasmids are shown in a representative gel image. lane 1: pGEM3Zf(−) construct with a clustered DNA damage site (uracil + 8-oxoG) and a mismatch (+21), lane 2: pGEM3Zf(−) construct without damage, but a XhoI site inserted at +21, lane 3: pGEM3Zf(−) construct without damage. The fraction of the intensity of the uncut pGEM3Zf(−) construct (3202 bp) to the intensity of all of the fragments (3202 bp + 1820bp + 1382 bp) represents the extent of the 8-oxoG strand used as a template (See text for further details). (**B**) Mismatch sites in the pGEM3Zf(−) construct. The numbers show the separation (in nt) from 8-oxoG within the bi-stranded cluster, with − and + indicating the 5′ and 3′ direction from 8-oxoG, respectively. The black arrow designates the direction of the replication fork from the replication origin (ori). (**C**) Mismatch sites in the pMW119f1(−) construct. The numbers show the separation (in nt) from 8-oxoG within the bi-stranded cluster, with − and + indicating the 5′ and 3′ direction from 8-oxoG, respectively. The black arrow designates the direction of the replication fork from the replication origin (ori).

To determine whether template strand preference is present around a DNA damage site, the 6-nucleotide (nt) XhoI recognition sequence (5′-CTCGAG-3′) was inserted within the uracil-containing strand of a plasmid as a strand-specific marker (Fig. 1A). A synthetic 8-oxoG lesion was inserted into the complementary strand opposite the uracil. After the plasmids were introduced into and propagated in *E. coli*, template strand preference was measured based on the fraction of the plasmid progeny with an uncut 8-oxoG stand, which lacked the XhoI sequence (a mismatch). In *E. coli*, a 6-nt mismatch is refractory to mismatch repair^26,27^, and strand synthesis past the mismatch site would either eliminate or retain the XhoI recognition sequence, depending on which strand is used as a template. The positions of the mismatch relative to the DNA lesion in plasmids pGEM3Zf(−)NAEPBNG and pMW119f1(−)NAEPBNG are shown in Fig. 1B, C, respectively. It is notable that uracil in a clustered DNA damage site is rapidly converted into an AP site and then subsequently processed to generate an SSB in *E. coli* cells^28^. In a previous study, little loss was observed for plasmid constructs with a bi-stranded clustered DNA damage site comprising uracil and 8-oxoG (uracil + 8-oxoG) in *E. coli* transformants, and enhanced mutagenic potential was found for the 8-oxoG within the cluster, implying that the repair of 8-oxoG within the cluster is compromised but the potential strand break (derived from uracil) is repaired or tolerated in *E. coli*^10^.

### Template strand preference around DNA lesions in a RecA- and PolI-proficient strain

**Figure 2:**
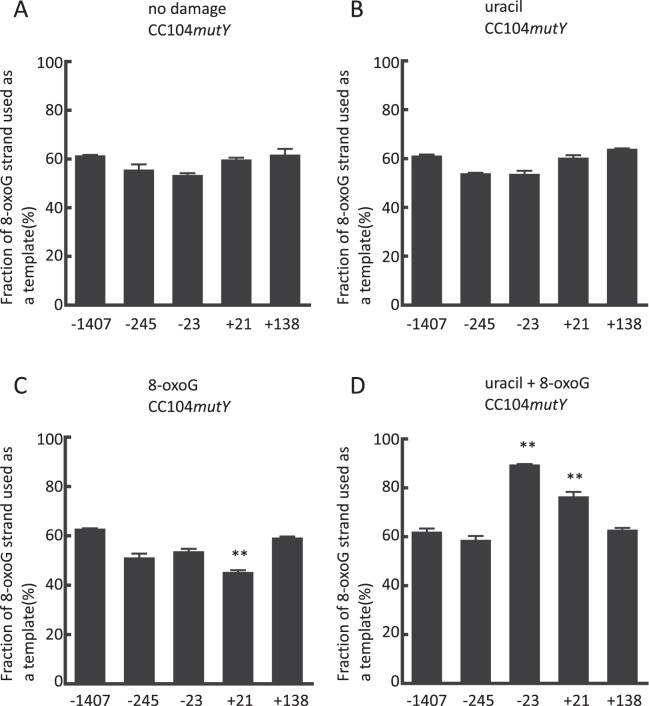
The fraction of the 8-oxoG strand used as a template when the pGEM3Zf(−) construct was propagated in CC104*mutY*. Panel A: no damage, Panel B: single uracil. Panel C: single 8-oxoG. Panel D: uracil + 8-oxoG bi-stranded clustered DNA damage. Along the horizontal axis, the positions of the mismatch are shown. Data represent the mean ± standard error (n = 3). Statistically significant differences between the fractions with and without DNA damage of the same mismatch site are indicated (**p < 0.01).

**Figure 3:**
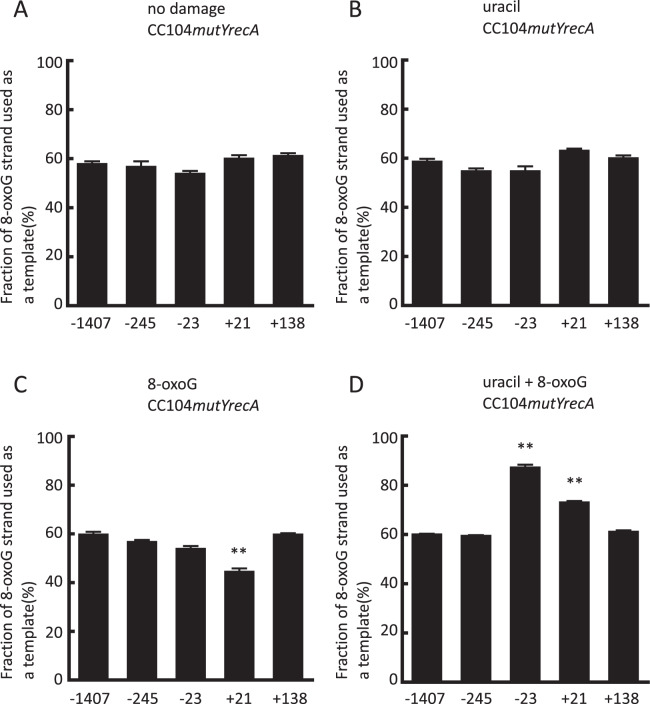
The fraction of the 8-oxoG strand used as a template when the pGEM3Zf(−) construct was propagated in CC104*mutYrecA*. Panel A: no damage, Panel B: single uracil. Panel C: single 8-oxoG. Panel D: uracil + 8-oxoG bi-stranded clustered DNA damage. Along the horizontal axis, the positions of the mismatch are shown. Data represent the mean ± standard error (n = 3). Statistically significant differences between the fractions with and without DNA damage of the same mismatch site are indicated (**p < 0.01).

**Figure 4:**
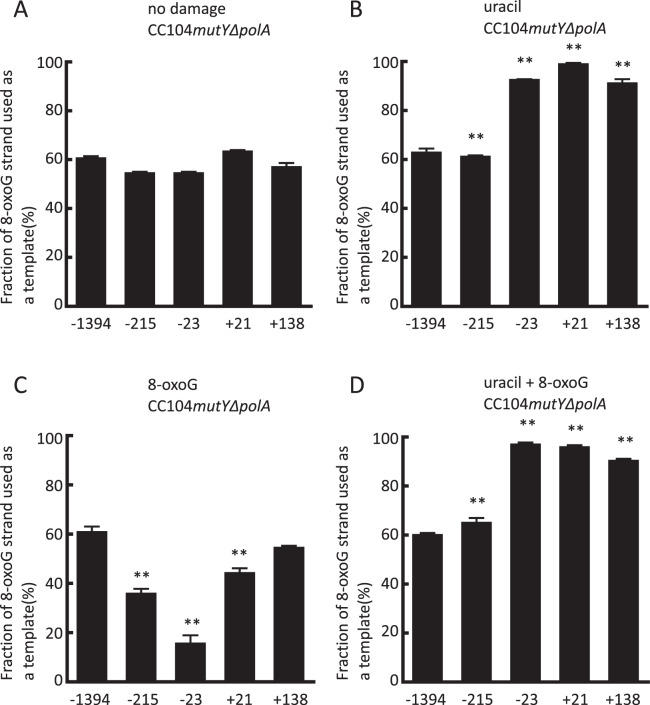
The fraction of the 8-oxoG strand used as a template when the pMW119f1(−) construct was propagated in CC104*mutY∆polA*. Panel A: no damage, Panel B: single uracil. Panel C: single 8-oxoG. Panel D: uracil + 8-oxoG bi-stranded clustered DNA damage. Along the horizontal axis, the positions of the mismatch are shown. Data represent the mean ± standard error (n = 3). Statistically significant differences between the fractions with and without DNA damage of the same mismatch site are indicated (**p < 0.01).

Using the plasmid pGEM3Zf(−)NAEPBNG, we found that the fraction of the template strand in undamaged plasmids deviated from the theoretical 50%, as was observed in previous experiments^26,29^, and that this fraction varied slightly among plasmids with mismatches at different sites (Figs. 2A, 3A, and 4A). The reason for these small deviations and variations among mismatched sites is unclear. In the present study, the strand preferences in the presence and in the absence of damage were compared at each mismatched site. In a RecA- and PolI-proficient strain, the fraction of the 8-oxoG strand used as a template did not differ regardless of the presence of a single uracil (U-1) for any mismatch sites (Fig. 2B). The fraction of the 8-oxoG strand was also unaffected by a single 8-oxoG, except for the mismatch at position +21, where the fraction was smaller than that of the undamaged construct (p < 0.01) (Fig. 2C). These results indicate that the repair of 8-oxoG has a longer patch size than that of uracil. The distinct repair patch sizes between uracil and 8-oxoG are likely attributable to the type of DNA lesion and/or the relative ratios of the participating BER enzymes^30,31^. Compared with single lesions, a bi-stranded clustered DNA damage site (uracil + 8-oxoG) had a distinct influence on the fraction of the 8-oxoG strand used as a template (Fig. 2D). At +21 and −23, the 8-oxoG strand was used much more frequently (p < 0.01) compared with fractions of the corresponding plasmids without any damage. We confirmed that the presence of a mismatch does not affect the enhanced mutagenic potential of the cluster^10^ (Supplementary Fig. S1).

### Template strand preference around DNA lesions in a RecA-deficient strain is similar to that in a RecA-proficient strain

The strand preference observed around a clustered DNA damage site may have resulted from replication fork reversal and/or homologous recombination. We next examined the fraction of the 8-oxoG strand used as a template in RecA-deficient cells to determine whether the absence of RecA affected the template strand preference. We found that the fractions with a single lesion in RecA-deficient cells were quite similar to those in RecA-proficient cells (Fig. 3B, C and Fig. 2B, C) and that, overall, the template strand preference observed around a clustered DNA damage site in the RecA-deficient strain was quite similar to that in the RecA-proficient strain (Fig. 3D and Fig. 2D). These results indicate that RecA is not primarily involved in forming a bias in template strand preference around a clustered DNA damage site. We found that the mutagenic potential of clustered DNA damage is remarkably higher than that of a single lesion in the presence of a mismatch in RecA-deficient cells (Supplementary Fig. S2), which is analogous to the case in RecA-proficient cells.

### The region of template strand preference around DNA lesions is larger in a PolI-deficient strain than in a PolI-proficient strain

Because we previously found that PolI is involved in suppressing mutagenesis within clustered DNA damage sites^13^, we next aimed to clarify the role of PolI in generating the strand preference around DNA lesions. Plasmid pGEM3Zf(−)NAEPBNG has a pMB1 replication origin and cannot propagate in a PolI-deficient background. Therefore, we used pMW119f1(−)NAEPBNG, which has a pSC101 replication origin. In our experiments, the template strand preferences in the region of the mismatches in pMW119f1(−) constructs were comparable to those in pGEM3Zf(−) constructs in the PolI-proficient cells (Fig. 2 and Supplementary Fig. S3). It should be noted, however, that the separations (in nt) of the mismatch from 8-oxoG were slightly different between the two plasmids (Fig. 1B, C). This was because identical oligonucleotides were used to generate the mismatch but the location of the oligonucleotide sequence differed between the two plasmids.

The presence of a single uracil dramatically increased the fractions of the 8-oxoG strand used as a template at −215, −23, +21 and +138, and up to over 90%, at −23, +21 and +138 in the PolI-deficient cells (Fig. 4B; p < 0.01), whereas the presence of a single 8-oxoG clearly decreased the fractions at −215, −23 and +21 (p < 0.01), and was lowest at −23 (<20%; Fig. 4C). With a clustered DNA damage site, the fractions of the 8-oxoG strand used as a template at −215, −23, +21 and +138 were again significantly elevated (p < 0.01) compared with those of the undamaged plasmids (Fig. 4D). These results indicate that the range of strand preference around DNA lesions in the PolI-deficient strain is much larger than that in the PolI-proficient strain. We confirmed that, in the PolI-deficient strain, the MF within the cluster was remarkably higher than that of a single lesion with any mismatch (Supplementary Fig. S4). Consistent with a previous study^13^, the MF within the clustered DNA damage site in the PolI-deficient strain was markedly higher than that in the PolI-proficient strain (Supplementary Fig. S4 and Fig. S5). We further found that the absence of Fpg in either the PolI-proficient or PolI-deficient cells did not alter the strand preference near the bi-stranded clustered DNA damage site (uracil + 8-oxoG) (Supplementary Figs. S6 and S3D, Fig. 4D), suggesting that the level of 8-oxoG removal by Fpg is, at most, quite low within the cluster.

## Discussion

Our results indicate that, in the vicinity of a bi-stranded clustered DNA damage site comprising uracil and 8-oxoG, the 8-oxoG strand is preferentially used as a template and that the strand preference likely plays an important role in enhanced mutagenic potential of 8-oxoG. The observed bias in the template strand was characteristic to the clustering of the lesions. Sites at −23 and +21 showed a strong preference for the 8-oxoG strand being used as the template, which revealed that sequences in both the 5′ and 3′ directions from uracil were replaced. Previous studies have shown that the decreased MF within the bi-stranded clustered DNA damage sites is more readily observed when the separation between the lesions is increased in the 3′ rather than the 5′ direction on the complementary strand of the mutagenic lesion^11,17^, which was consistent with the present finding that the level of strand replacement in the 5′ direction from uracil (+21) was lower compared with that in the 3′ direction (−23).

Previous findings indicate that, in the case of a bi-stranded cluster containing an 8-oxoG on one strand and another lesion (uracil/ 2-deoxyribonolactone/DHT/Tg) on the complementary strand, the repair of the SSB resulting from lesion processing on the complementary strand is retarded and the SSB persists until replication^10–12,14^. In canonical BER or single strand break repair in *E. coli*, the 3′ incision downstream from base damage or single strand break is most likely generated by the 5′ nuclease activity of PolI^7,31^, but, to our knowledge, 5′ incision several tens of nt upstream from the damage has not been reported. These results, together with our present observations that the strand is replaced in both 5′ and 3′ directions from the lesion, support the notion that, within the clustered DNA damage site, BER is compromised and another processing pathway deals with the persistent SSB, thus leading to the characteristic template strand preference observed. As the transformation efficiency of plasmids with clustered DNA damage sites is comparable to that of plasmids with undamaged constructs^10,28^, the SSB processing at a bi-stranded clustered DNA damage site (uracil + 8 G) does not lead to plasmid linearization.

A persistent SSB would impede the progression of the replication fork. We found that the strand preference in a RecA-deficient strain was essentially the same as that in a RecA-proficient strain. This indicates that RecA does not play a major role in tolerating the persistent SSB and in generating the template strand preference in *E. coli*; at least, not under our experimental conditions. Although RecA was found to influence the template strand preference flanking a 1,*N*^6^-ethenodeoxyadenosine adduct when using plasmid-based assays^29^, Fuchs’ group demonstrated a replication-blocking type of lesion, such as an *N*-2-acetylaminofluorene adduct, is tolerated by the RecA-dependent homology-directed gap repair within genomic DNA but not within transfected plasmid DNA^32,33^. They concluded that RecA is unable to participate in the damage tolerance of plasmid DNA as the replication fork gets uncoupled. As there was no template strand preference ~1,400 nt away from the clustered damage site in any of the strains used, we consider it unlikely that uncoupling of the fork took place in the present study. Another study demonstrated that the inactivation of RecA, RecF or RecG does not affect the survival of transformed plasmids with replication-blocking lesions (i.e. pyrimidine dimers) in a nucleotide-repair-deficient background^34^. The results led the authors to conclude that lesions in the transformed plasmid DNA are repaired within the time gained by an inefficient replication, thus obviating the need for the RecA-dependent tolerance pathway. Further investigations are required to clarify whether the reduced role of RecA in the present study was due to the type of lesion (an SSB rather than a replication block) or the transformation of the plasmid.

Unlike the case of RecA, the absence of PolI led to a markedly greater range of preferential strand synthesis encompassing a DNA lesion. Even the presence of either a single uracil or a single 8-oxoG strongly affected the strand preference around the lesion in the 5′ direction as well as the 3′ direction, although this effect seemed less enhanced in the 3′ direction. Our result is consistent with a previous study that indicated a similar expanded region of strand replacement from a single, MutY-removed adenine in a PolI-deficient strain^35^. We further found that the length of strand synthesis in the region of a bi-stranded clustered DNA damage site was quite similar to that for a single uracil in PolI-deficient cells. This may not be so surprising as neither the SSB created within a cluster nor from a single uracil is likely subjected to BER. An as yet unknown SSB repair or SSB tolerance pathway seems to operate in cells even when PolI is absent.

**Figure 5:**
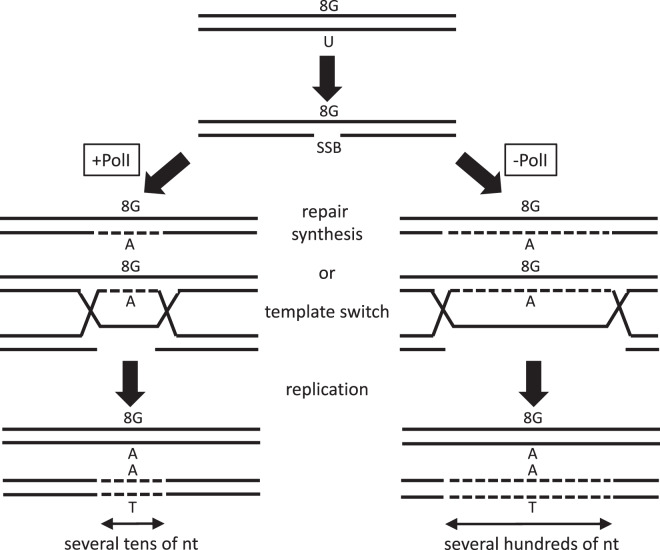
Possible mechanisms generating the strand bias around a bi-stranded cluster containing uracil and 8-oxoG. When PolI is present, the persistent SSB generated within the cluster is either processed by repair synthesis before replication or by template switching during replication with a patch size of several tens of nt. The strand synthesis is most likely a process unrelated to BER or NER (see Discussion). When PolI is absent, the persistent SSB generated by lesion clustering is either processed, with the use of polymerase(s) other than PolI, by repair synthesis before replication or by template switching during replication with a patch size of several hundreds of nt.

When an obstacle to replication leads to damage avoidance, a body of evidence points towards the occurrence of a template switch, which commonly involves recombination^22,25^. Our results suggest that, in the presence of PolI, a RecA-independent template switching pathway, which operates during replication fork stalling, fork regression or fork collapse, could have potentially mediated the strand synthesis involving incorporation of adenine opposite 8-oxoG and elimination of a nearby mismatch (Fig. 5, pathway A). Growing evidence supports the idea that the presence of a nick/SSB initiates homology-dependent repair not only via the canonical RecA- or Rad51-dependent strand invasion pathways but via an alternative pathway independent of RecA or Rad51 function^36,37^. An alternative possibility regarding the emergence of strand preference is the repair synthesis of the SSB before replication. For instance, nucleotide excision repair (NER), in which the repair synthesis is mediated by PolI, could be responsible for the biased use of a template strand. SSBs derived from an AP site by Endonuclease III or Endonuclease IV are indeed known to be a substrate of NER^38^. However, we consider the involvement of NER to be unlikely, as the region of strand bias observed in the PolI-proficient strain (~50 nt) was clearly larger than the repair patch length of NER in *E. coli*, where the majority of the patch lengths are in the range of 12–20 nt^39^. Consistent with our view that NER is unrelated to the occurrence of the strand bias, the levels of strand preference at sites −23 and +21 in either UvrA- or UvrC-deficient strain, which is NER deficient, were comparable with the respective levels in an NER-proficient strain (Supplementary Figs S7 and Fig. S3D). We consider that the repair/tolerance pathway of a persistent SSB is likely independent of whether the SSB is on the leading or the lagging strand template, as the direction of the replication fork does not affect the MFs of 8-oxoG placed within or nearby bi-stranded clustered damage sites^13,18^. Elucidation of the underlying mechanism(s) of strand preference awaits further investigation. When PolI is absent, a larger repair tract is required for the processing of the persistent SSB (Fig. 5, pathway B). DNA polymerases other than PolI likely synthesized the DNA and are most probably involved in the repair or avoidance of a persistent SSB with a relatively large tract.

In summary, based on our plasmid-based assay, we found that, in the region of a clustered DNA damage site comprising uracil and 8-oxoG, the strand harbouring a mutagenic lesion (8-oxoG) is preferentially used as the template. The region of such strand preference is in the order of several tens of nt. The length of strand synthesis is determined by the function of PolI but is unaffected by RecA under our experimental conditions. Although the underlying mechanism for this strand preference has not yet been clarified, we suggest that the process is unrelated to canonical BER or NER and is of significant importance for coping with a persistent SSB *in vivo*.

## Materials and Methods

### Bacterial strains

Strain CC104*mutY::Tet* was provided by Dr. Q-M. Zhang-Akiyama (Kyoto University), strain AJW377 harbouring *recA::Cm* was a gift from Dr. A. J. Wolfe (Loyola University Chicago), and strain CS5539 harbouring *uvrA::Cm* was provided by Dr. N. Goosen (Leiden University). *recA::Cm* from AJW377 and *uvrA::Cm* from CS5539 were transferred to CC104*mutY* through P1 transduction, to construct CC104*mutYrecA* and CC104*mutYuvrA*, respectively. Strains CC104*fpgmutY*, CC104*mutYΔpolA* and CC104*fpgmutY∆polA* were also constructed by P1 transduction, as reported previously^13^. To construct strain CC104*mutYuvrC*, the *uvrC* gene of CC104*mutY* was replaced with a kanamycin-resistance cassette by the Lambda Red protocol^40^. All of the strains in the present study lacked the wild-type *mutY* gene, as these strains enable detailed monitoring of the reparability of 8-oxoG^10,13^.

### Oligonucleotides and plasmids

The 5′-phosphorylated oligonucleotides were purchased from Tsukuba Oligo Service (Tsukuba, Japan) (Table 1). NAEP and U-1 are identical except for the presence of uracil in U-1, and BNG and 8G are identical except for the 8-oxoG in 8G. NAEP and BNG are complementary sequences, and NAEP + BNG double-stranded oligonucleotides were inserted between the EcoRI and PstI sites of pGEM3Zf(−) to construct pGEM3Zf(−)NAEPBNG. Plasmid pMW119f1(−)NAEPBNG was constructed by replacing the HindIII–NdeI fragment of pMW119 with the HindIII–NdeI fragment of pGEM3Zf(−)NAEPBNG. Oligonucleotides X-1407/X-1394, X-245/X-215, NAEPX-23, U-1EPX-23, NAEPX + 21, U-1EPX + 21 and X + 138 contained the 6-nt XhoI recognition sequence (5′-CTCGAG-3′), which formed a mismatch in plasmid pGEM3Zf(−)NAEPBNG. Analogously, X-1407/X-1394, X-245/X-215, 119NAEPX-23, 119U-1EPX-23, NAEPX + 21, U-1EPX + 21 and X + 138 were used to create a mismatched site in pMW119f1(−)NAEPBNG.

**Table 1:** Oligonucleotides used in this study.

NAEP	5′AATTCTCTTAGTCAGGAATATGTCTCTATGCTGGGAGCAAAGGCTGCA3′
BNG	5′GCCTTTGCTCCCAGCATAGAGACATATTCCTGACTAAGAG3′
U-1	5′AATTCTCTTAGTCAGGAATATG**U**CTCTATGCTGGGAGCAAAGGCTGCA3′
8G	5′GCCTTTGCTCCCAGCATAGA**8G**ACATATTCCTGACTAAGAG3′
X-1407/X-1394	5′GATGCTTTTCTGTGACTGGTGAGTCTCGAGACTCAACCAAGTCATTCTGAGAAT3′
X-245/X-215	5′CAGTCGGGAAACCTGTCGTGCCAGCTCGAGCTGCATTAATGAATCGGCCAACGC3′
X-23	5′AATTCTCTTAGTCAGGAATATGTCTCTATGCTGGGAGCAAAGGCTGCTCGAGCAGGCATGCAAGCTTGAGTATTCT3′
119X-23	5′AATTCTCTTAGTCAGGAATATGTCTCTATGCTGGGAGCAAAGGCTGCTCGAGCAGGCATGCAAGCTTGGCGTAATC3′
X-23U-1	5′AATTCTCTTAGTCAGGAATATG**U**CTCTATGCTGGGAGCAAAGGCTGCTCGAGCAGGCATGCAAGCTTGAGTATTCT3′
119X-23U-1	5′AATTCTCTTAGTCAGGAATATG**U**CTCTATGCTGGGAGCAAAGGCTGCTCGAGCAGGCATGCAAGCTTGGCGTAATC3′
X + 21	5′TAATACGACTCACTATAGGGCGAACTCGAGTTCTCTTAGTCAGGAATATGTCTCTATGCTGGGAGCAAAGGCTGCA3′
X + 21U-1	5′TAATACGACTCACTATAGGGCGAACTCGAGTTCTCTTAGTCAGGAATATG**U**CTCTATGCTGGGAGCAAAGGCTGCA3′
X + 138	5′CGGGCCTCTTCGCTATTACGCCAGCTCGAGCTGGCGAAAGGGGGATGTGCTGCA3′

U in the oligonucleotide sequence represents uracil, while 8G indicates 8-oxoG. The six nucleotide-long XhoI recognition sequence (5′-CTCGAG-3′), which forms a mismatch in plasmid pGEM3Zf(−)NAEPBNG and pMW119f1(−)NAEPBNG, is shown by single underline. The number after X in the oligonucleotide harbouring a mismatch denotes the separation of the mismatch in nt from the 8-oxoG, and − and + indicate the 5′ and 3′ direction from 8-oxoG, respectively. 119X−23 and 119X−23U-1 are oligonucleotides that anneal with pMW119f1(−)NAEPBNG. Alw26I recognition sequences are shown by double underline. When U-1 is annealed with 8G to form a bi-stranded uracil + 8-oxoG cluster, uracil is located 1 nt 5′ from 8-oxoG on the complementary strand.

### Construction of a plasmid with DNA damage and a mismatch

Plasmids with DNA damage and a mismatch were constructed according to the method described previously^26^, with slight modifications. In brief, to the cultures of CJ236 harbouring pGEM3Zf(−)NAEPBNG or pMW119f1(−)NAEPBNG grown in the presence of uridine (75 µg/mL), VCM13 helper phages were added and phage particles were precipitated overnight. Uracil-containing circular single-stranded (ss) DNA was extracted from the pelleted phage particles with lysis buffer (10 mM MOPS, pH 6.5, 1% Triton X-100, 500 mM guanidine-HCl) and a Qiagen HiSpeed Plasmid Midi kit (Qiagen, Tokyo, Japan). The oligonucleotides (50 pmol) were annealed with 20 pmol of uracil-containing circular ssDNA. The 1^st^ strand was synthesized from the annealed primer in a reaction volume of 540 µL in the presence of 50 mM Tris–HCl, pH 7.5, 5 mM MgCl_2_, 1 mM DTT, 0.5 mM ATP, 0.185 mM dNTPs, 5% PEG (average MW 8,000), 0.2 U pyrophosphatase (New England Biolabs, Tokyo, Japan), 50 U native T7 DNA polymerase (New England Biolabs) and 15 U T4 DNA ligase (Thermo Fisher Scientific, Tokyo, Japan) at 33 °C for 30 min, followed by heat inactivation of the enzymes at 75 °C for 20 min. After digestion of the uracil-containing template strand at 37 °C for 1 h with 3U UDG, 50 U Exonuclease I and 50U Exonuclease III (all from New England Biolabs), the resultant ss circular 1^st^ strand was purified with a QIAquick Gel Extraction Kit (Qiagen). Twenty pmol of the 5′-phosphorylated primer was annealed to the 1^st^ strand, and the 2^nd^ strand was synthesised in the same reaction mixture used for 1^st^ strand synthesis but in a reaction volume of 270 µl. The final product (circular dsDNA) was ethanol-precipitated and gel-purified with a QIAquick Gel Extraction Kit.

### Transformation and analysis of amplified plasmids

The constructed plasmids (6.6 ng) were electroporated into *E. coli* cells with a Bio-Rad *E.coli* pulser set at 1.8 kV, 200 Ω and 25 µF. Plasmids from the overnight culture were extracted with a QIAprep Spin Miniprep Kit (Qiagen). To quantitate the fraction of the strand used as a template, plasmids were first linearized with ScaI, except for plasmids synthesized with X-1407/X-1394 (which were linearized with HindIII), and the linearized fragments were further digested with XhoI. The complete digestion with XhoI was confirmed by independent digestion with another restriction endonuclease, of which the recognition sequence was located opposite the XhoI recognition site in the constructed plasmid. After digestion with the restriction enzymes, plasmids were electrophoresed in an agarose gel, and the relative intensities of the ethidium bromide-stained DNA fragments were measured with a gel imaging system (FluoroChem Imager, ProteinSimple, Tokyo, Japan). The fraction of the strand used as a template was estimated based on the intensity of the uncut fragment relative to the intensity of all of the fragments (both cut and uncut fragments). Transformation was repeated three times for each plasmid construct, and the statistical significance of the differences in the fractions of uncut fragments between the plasmids was determined with the two-tailed t-test.

## Supplementary information

Supplementary information.

## Data Availability

All the data generated or analysed during this study are included in this article and its supplementary information files.
