# Supplementary material for: Strand with mutagenic lesion is preferentially used as a template in the region of a bi-stranded clustered DNA damage site in Escherichia coli
Source: Sci Rep. 2020 Jun 16;10:9737. doi: 10.1038/s41598-020-66651-0 (PMC7297740; doi:10.1038/s41598-020-66651-0)
Supplement: Supplementary file 1 — Supplementary information. [file 41598_2020_66651_MOESM1_ESM.docx]

**Supplementary information**

**Strand with mutagenic lesion is preferentially used as a template in the region of a bi-stranded clustered DNA damage site in *Escherichia coli***

Naoya Shikazono* and Ken Akamatsu

Institute for Quantum Life Science, National Institutes for Quantum and Radiological Science and Technology

*Corresponding author: Naoya Shikazono

email: shikazono.naoya@qst.go.jp

Supplementary Fig. S1

Mutation frequencies of damaged sites in CC104*mutY* transformed with pGEM3Zf(−) constructs. Along the horizontal axis, the positions of the mismatch are shown. Black bar, no damage; white bar, single uracil; grey bar, single 8-oxoG; bar with stripes, uracil + 8-oxoG cluster. Uracil and 8-oxoG are both placed within the Alw26I recognition sequence (Table 1), and the mutation frequency of the damage sites was determined based on the intensity of the undigested fraction of the plasmids treated with Alw26I (Pearson, C. G., Shikazono, N., Thacker, J. & O'Neill, P. *Nucleic Acids Res* **32**, 263-270 (2004)). Data represent the mean ± standard error (n = 3).

Supplementary Fig. S2

Mutation frequencies of damaged sites in CC104*mutYrecA* transformed with pGEM3Zf(−) constructs. Along the horizontal axis, the positions of the mismatch are shown. Black bar, no damage; white bar, single uracil; grey bar, single 8-oxoG; bar with stripes, uracil + 8-oxoG cluster. Uracil and 8-oxoG are both placed within the Alw26I recognition sequence (Table 1), and the mutation frequency of the damage sites was determined based on the intensity of the undigested fraction of the plasmids treated with Alw26I (Pearson, C. G., Shikazono, N., Thacker, J. & O'Neill, P. *Nucleic Acids Res* **32**, 263-270 (2004)). Data represent the mean ± standard error (n = 3).

Supplementary Fig. S3

The fraction of the 8-oxoG strand used as a template when the pMW119f1(−) construct was propagated in CC104*mutY∆polA.* Panel A: no damage, Panel B: single uracil. Panel C: single 8-oxoG. Panel D: uracil + 8-oxoG bi-stranded clustered DNA damage. Along the horizontal axis, the positions of the mismatch are shown. Data represent the mean ± standard error (n = 3). Statistically significant differences between the fractions with and without DNA damage of the same mismatch site are indicated (**p < 001).

Supplementary Fig. S4

Mutation frequencies of damaged sites in CC104*mutY∆polA* transformed with pMW119f1(−) constructs. Along the horizontal axis, the positions of the mismatch are shown. Black bar, no damage; white bar, single uracil; grey bar, single 8-oxoG; bar with stripes, uracil + 8-oxoG cluster. Uracil and 8-oxoG are both placed within the Alw26I recognition sequence (Table 1), and the mutation frequency of the damage sites was determined based on the intensity of the undigested fraction of the plasmids treated with Alw26I (Pearson, C. G., Shikazono, N., Thacker, J. & O'Neill, P. *Nucleic Acids Res* **32**, 263-270 (2004)). Data represent the mean ± standard error (n = 3).

Supplementary Fig. S5. Mutation frequencies of damaged sites in CC104*mutY* transformed with pMW119f1(−) constructs. Along the horizontal axis, the positions of the mismatch are shown. Black bar, no damage; white bar, single uracil; grey bar, single 8-oxoG; bar with stripes, uracil + 8-oxoG cluster. Uracil and 8-oxoG are both placed within the Alw26I recognition sequence (Table 1), and the mutation frequency of the damage sites was determined based on the intensity of the undigested fraction of the plasmids treated with Alw26I (Pearson, C. G., Shikazono, N., Thacker, J. & O'Neill, P. *Nucleic Acids Res* **32**, 263-270 (2004)). Data represent the mean ± standard error (n = 3).

Supplementary Fig. S6

The fraction of the 8-oxoG strand used as a template when the pMW119f1(−) construct harbouring a bi-stranded clustered DNA damage site (uracil + 8G) was propagated in CC104*fpgmutY* and CC104*fpgmutY∆polA*. The numbers along the horizontal axis denote the position of the mismatch. Data represent the mean ± standard error (n = 3).

Supplementary Fig. S7. The fraction of the 8-oxoG strand used as a template when the pMW119f1(−) construct harbouring a bi-stranded clustered DNA damage site (uracil + 8G) was propagated in CC104*mutYuvrA* or CC104*mutYuvrC*. The numbers along the horizontal axis denote the position of the mismatch. Data represent the mean ± standard error (n = 3).
